# Establishment of an Australian National Master Linkage Key (NMLK) between AIHW Health Linkage spine (HLS) and Jurisdictional Master Linkage Keys (MLK) - NSW example

**DOI:** 10.23889/ijpds.v9i5.1648

**Published:** 2024-09-18

**Authors:** Elena Ougrinovski, Clinton Paine, Natalie Cooper, Pooja Nair, Michael Smith, Rama Devi Voruganti, Merran Smith, Felicity Flack

**Affiliations:** 1 Australian Institute of Health and Welfare; 2 Centre for Health Record Linkage; 3 Population Health Research Network

## Objectives

This paper describes a Population Health Research Network project establishing the NMLK as a national linkage system utilising an enduring linkage between national and jurisdictional linkage spines. It used keys from existing enduring linkages within each participating jurisdictional Data Linkage Unit (DLU). The process is demonstrated with reference to an enduring map between AIHW Health Linkage Spine (HLS) and Centre for Health Record Linkage (CHeReL) MLK.

## Approach

The NMLK has jurisdictional sections. The NSW section was established in four steps.

1. CHeReL provided data from its regularly updated MLK for the NMLK project authorised by existing project ethics approval.
2. AIHW linked NSW MLK subset to HLS and the de-identified linkage map and report were sent to CHeReL for review.
3. CHeReL reviewed the results, particularly the additional duplicates identified through the linkage process. The agreed links form the AIHW-NSW section of NMLK quarantined for a year.
4. The annual update, scheduled for later in 2024, will focus only on linking the new and unlinked individuals in both datasets.

## Results

The NSW cohort was supplied with almost 50 million records, comprising over 14 million unique person ids. Of these, 12 million were matched with reasonable confidence to HLS, or 84.4% of the total (or around 97% if we exclude NSW records with missing personal information and around 140,000 individuals out of scope of HLS.)

## Conclusions and Implications

Establishment of the NMLK enabled effective production of high-quality enduring linkage maps used across multiple cross-jurisdictional projects while minimising the privacy risks.

